# Facile Synthesis of Water-Soluble Rhodamine-Based Polymeric Chemosensors *via* Schiff Base Reaction for Fe^3+^ Detection and Living Cell Imaging

**DOI:** 10.3389/fchem.2022.845627

**Published:** 2022-02-28

**Authors:** Xiaoyong Qiu, Jun Huang, Ning Wang, Kaijie Zhao, Jiwei Cui, Jingcheng Hao

**Affiliations:** ^1^ Key Laboratory of Colloid and Interface Chemistry of the Ministry of Education, School of Chemistry and Chemical Engineering, Shandong University, Jinan, China; ^2^ Key Laboratory of High Efficiency and Clean Mechanical Manufacture of the Ministry of Education, Center for Advanced Jet Engineering Technologies (CaJET), School of Mechanical Engineering, Shandong University, Jinan, China

**Keywords:** Fe^3+^ detection, fluorescence probe, rhodamine, Schiff base, cell imaging

## Abstract

Quantitative and accurate determination of iron ions play a vital role in maintaining environment and human health, but very few polymeric chemosensors were available for the detection of Fe^3+^ in aqueous solutions. Herein, a water-soluble rhodamine-poly (ethylene glycol) conjugate (DRF-PEG), as a dual responsive colorimetric and fluorescent polymeric sensor for Fe^3+^ detection with high biocompatibility, was first synthesized through Schiff base reaction between rhodamine 6G hydrazide and benzaldehyde-functionalized polyethylene glycol. As expected, the introduction of PEG segment in DRF-PEG significantly improved the water solubility of rhodamine derivatives and resulted in a good biosensing performance. The detection limit of DRF-PEG for Fe^3+^ in pure water is 1.00 μM as a fluorescent sensor and 3.16 μM as a colorimetric sensor at pH 6.5. The specific sensing mechanism of DRF-PEG toward Fe^3+^ is proposed based on the intramolecular charge transfer (ICT) mechanism, in which the O and N atoms in rhodamine moiety, together with the benzene groups from benzaldehyde-modified PEG segment, participate in coordination with Fe^3+^. Furthermore, DRF-PEG was applied for the ratiometric imaging of Fe^3+^ in HeLa cells and showed the potential for quantitative determination of Fe^3+^ in fetal bovine serum samples. This work provides insights for the design of water-soluble chemosensors, which can be implemented in iron-related biological sensing and clinical diagnosis.

## Introduction

Iron ion is one of the most abundant transition metal ions in the human body and plays a vital role in biological metabolism (Guo et al., 2015; Liu et al., 2017; Fan et al., 2019). The deficiency in iron will lead to the lack of iron-related proteins and cause anemia, cancer, or other health problems (Park and Lee, 2020), while excess iron can inhibit the absorption of nutrients and cause irreversible damage to neurological systems and human organs, leading to serious diseases, such as Alzheimer’s disease, hepatic fibrosis or even death (Nayab and Shkir, 2017; Lin et al., 2020). The determination of Fe^3+^ in natural water or human body fluid is of great importance to human health and biological environment. Among the numerous detection assays, such as atomic absorption (Frankowski et al., 2010), mass spectrometry (Jia et al., 2018), Raman scattering (Song et al., 2020), and so on, optical spectroscopy methods combined with molecular sensors have attracted much attention for the characteristics of easy operation, high sensitivity, and high throughput (Cao et al., 2014; Jin et al., 2018; Jung et al., 2019). Many chemosensors for Fe^3+^ have been developed, but very few can be directly applied in water or biological samples due to their intrinsic hydrophobicity and cytotoxicity (Carter et al., 2014; Hou et al., 2017). Furthermore, background interference from the testing environment, such as bacteria or proteins in biosamples, may result in a significant reduction of signal-to-noise ratio and lead to detection failure (Zhang et al., 2019). Therefore, fluorescent chemosensors with excellent water solubility and biocompatibility that can be applied in complex environmental or biological systems are highly desirable.

Due to the excellent optical properties and unique switching nature of spirocyclic structures, rhodamine derivatives are widely employed for detecting metal ions (Song Y. et al., 2019; Jiao et al., 2018; Wu et al., 2018). The sensing mechanism of rhodamine sensors is based on the structural transformation from spirolactam ring to open-loop state when combined with specific ions, releasing strong fluorescent signals. Most rhodamine-derived sensors can only work in organic solvent or organic solvent-water mixtures owing to their inherent poor water solubility, which greatly inhibits their applications in practical biological and environmental systems (Zhang Y. et al., 2020; Geng et al., 2017; Choudhury et al., 2020; Shi et al., 2016). Many efforts have been devoted to inventing fluorescent sensors with good water solubility and biocompatibility, which can be achieved by introducing water-soluble carbohydrates (Chen and Fang, 2018) or hydrophilic polymer segments (Li et al., 2015; Li et al., 2016; Geng et al., 2017; Rong et al., 2017; Wu et al., 2017) into rhodamine fluorophores.

Because of the excellent biocompatibility and hydrophilicity, poly (ethylene glycol) (PEG) is commonly used in biomedical research (Liu et al., 2015). By virtue of the antifouling properties of PEG toward proteins, it is expected that the detection performance of PEG-derived polymeric sensors would be improved and the background interference from biological contamination should be reduced (Liu et al., 2015). A few water-soluble polymeric sensors obtained by combining PEG segment and rhodamine moiety have been designed for the detection of Cu^2+^, Hg^2+^, and Al^3+^ (Li et al., 2016; Geng et al., 2017; Li et al., 2018). However, the rhodamine-PEG-conjugated polymeric sensors, which can be applied in detecting Fe^3+^ in pure aqueous solutions with high biocompatibility, have not been reported. This work aims to develop a highly water-soluble rhodamine-PEG-conjugated polymeric sensor for Fe^3+^ and evaluate its potential applications in biological systems.

Herein, rhodamine 6G hydrazide (Rh) is selected as the fluorescent/colorimetric sensing receptor, and PEG segment is chosen as the hydrophilic segment. The polymeric sensor (DRF-PEG) containing both PEG chain and rhodamine moiety is synthesized through a facile Schiff base reaction between the amine group (−NH_2_) of Rh and the aldehyde group (−CHO) of di-benzaldehyde terminated poly (ethylene glycol) (DF-PEG)*.* The rhodamine moiety of DRF-PEG serves as a sensing fluorophore in detecting metal ions, while the PEG segment improves the sensor’s water solubility and biocompatibility. DRF-PEG is highly sensitive and selective for Fe^3+^ in water with dual-responsive fluorescent and colorimetric response. MTT assay suggests that DRF-PEG possesses low cytotoxicity and good biocompatibility, which can be utilized for intracellular imaging. Furthermore, the detection performance of DRF-PEG for Fe^3+^ is evaluated in fetal bovine serum. This work provides a facial and typical model for designing polymeric sensors with high biocompatibility and water solubility, which can be applied in living cell imaging or metal ion detection in biological systems.

## Experimental Section

### Materials

Rhodamine 6G (95%), 1,3-dicyclohexylcarbodiimide (DCC, 99%), N,N-dimethylpyridin-4-amine (DMAP, 99%), and 4-carboxybenzaldehyde (98%) were obtained from Aladdin Chemistry Co. Ltd. (Shanghai, China). Polyethylene glycol (PEG, *M*_W_ 4,000 Da), hydrazine monohydrate (50%), silver nitrate, chloride salts of K^+^, Na^+^, Mg^2+^, Ca^2+^, Cu^2+^, Hg^2+^, Co^2+^, Cd^2+^, Zn^2+^, Ni^2+^, Mn^2+^, Al^3+^, Cr^3+^, and Fe^3+^ were purchased from Sinopharm Chemical Reagent Co. Ltd. MTT (thiazolyl blue tetrazolium bromide) was obtained from Thermo Fisher Scientific (China). Ultrapure water (18.2 MΩ cm) was obtained from the Milli-Q system and applied throughout the experiment. Other solvents and chemicals were of analytical reagent grade and used without further treatment.

### Instrumentation

^1^H NMR spectra were obtained on a Bruker Advance 400 spectrometer (Germany). FT-IR spectra were obtained on a TENSOR II FTIR routine spectrometer from Bruker (Germany) after pelleting samples with KBr. The UV-Vis absorption measurement was carried out on a Hitachi U-4100 UV/Vis/NIR spectrometer (Japan). Fluorescence spectra were performed on a FluoroMax-4 high efficiency integrated fluorescence spectrometer (Horiba. United States), and each measurement was repeated three times. The pH of the solution was measured by a Mettler Toledo SevenCompact pH meter S210. Confocal laser scanning microscopy (CLSM) images were recorded on a Leica TCS SP8 lighting confocal microscope (Germany).

### Synthesis

Rhodamine 6G hydrazide was prepared through the reduction of rhodamine 6G by hydrazine monohydrate using the reported procedure (Yang et al., 2002).

### Synthesis of Di-Benzaldehyde Terminated Poly (Ethylene Glycol)

DF-PEG was synthesized by following the previously reported method (Zhang et al., 2011; Yan et al., 2017). Under a nitrogen atmosphere, PEG_4000_ (8.0 g, 2.0 mmol) in dry THF was mixed with 4-formylbenzoic acid (0.90 g, 6.0 mmol) and DMAP (0.09 g). After complete dissolution of all the compounds by sonication, DCC (1.65 g) in 50 ml of THF was added. The mixture was thoroughly stirred at 25°C for 48 h and then filtered. Filtrates were dried through vacuum rotary evaporation and precipitated by ethyl ether three times. The obtained precipitate was dried under vacuum for 2 days to give DF-PEG as a white waxy solid. Yield: 6.4 g (75%). ^1^H NMR (400 MHz, CDCl_3_) *δ*: 3.40–3.90 (m, 360 H; −OCH_2_CH_2_–), 4.48–4.53 (m, 4 H; −COOCH_2_−), 7.95 (d, *J* = 8.4 Hz, 4 H; −ArH), 8.21 (d, *J* = 8.4 Hz, 4 H; −ArH), 10.10 (s, 2 H; −ArCHO).

### Synthesis of Polymeric Sensor Containing Both PEG Chain and Rhodamine Moiety

The solution of R6G hydrazide (0.11 g, 0.25 mM) in 20 ml of acetone was slowly added into 30 ml of acetone with DF-PEG (0.53 g, 0.13 mM). After refluxed at 58°C for 8 h and cooled overnight, the mixture was then filtered. The obtained filtrate was concentrated under vacuum to give DRF-PEG as a purple solid. Yield: 1.0 g (80%). ^1^H NMR (400 MHz, DMSO-*d*_*6*_) δ: 1.20 (t, J = 7.2 Hz, 12 H; −CCH_3_), 1.85 (s, 12 H; −ArCH_3_), 3.06–3.17 (m, 8 H; −NCH_2_−), 3.40–3.80 (m, 360 H; −OCH_2_CH_2_−), 4.40–4.46 (m, 4 H; −COOCH_2_−), 5.01 (t, *J* = 5.2 Hz, 4 H; −NH−), 6.16 (s, 4 H; −ArH), 6.21 (s, 4 H; −ArH), 6.94–6.99 (m, 2 H; −ArH), 7.48–7.53 (m, 4 H; −ArH), 7.76–7.81 (m, 2 H; −ArH), 8.06 (d, J = 8.4 Hz, 4 H; −ArH), 8.16 (d, *J* = 8.4 Hz, 4 H; −ArH), 10.12 (s, 2 H; −NCH−).

### Spectroscopic Study

Stock solutions of Fe^3+^ and other metal ions (1.0 × 10^–3^ mol/L) were prepared by dissolving the related inorganic salts in Milli-Q water for the fluorescence and absorption experiment. Stock solution of DRF-PEG was prepared in Milli-Q water with a concentration of 1.0 mg/ml. All the spectral experiments were conducted in an aqueous solution except for the absorption test related to Rh because of its poor water solubility. For the absorption and fluorescence selectivity experiment, DRF-PEG solution (0.1 mg/ml) was mixed with different metal ions (10^–4^ M). For the sensitivity experiment, DRF-PEG solution (0.1 mg/ml) was mixed with different concentrations of Fe^3+^. The fluorescence emission spectra of DRF-PEG were collected from 520–680 nm with an excitation wavelength of 500 nm. The quartz cuvette with a 1-cm path length was used for the absorption and emission studies. For the pH test, the pH of the tested solution was adjusted by using 0.1 mol/L of HCl, and 0.1 mol/L of NaOH solutions at 25°C.

### Cytotoxicity Investigation

The cytotoxicity of DRF-PEG was evaluated by a standard MTT assay on HeLa cells (human cervical carcinoma cells) obtained from Shanghai EK-Bioscience Biotechnology Co., Ltd.

HeLa cells were seeded at a density of 1 × 10^4^ cells/well and cultured in DMEM medium with 1% antibiotic and 10% FBS (fetal bovine serum). After cell attachment, the cells were incubated with different concentrations of DRF-PEG (0, 6.25, 12.5, 25, 50, 100, 125, 250, 500 μg/ml) and maintained at 37°C under 5% CO_2_ for 24 h. After washing with PBS buffer three times, fresh culture medium containing MTT (10 μl, 5 mg/ml) was added and incubated for another 4 h. Then the culture medium was removed and washed with PBS buffer. The samples were then dissolved in 150 μl of DMSO and shook for 10 min. Compared with the control sample, the cell viability was estimated through the optical density of the mixture at 450 nm.

### Living Cell Imaging

HeLa cells (1 × 10^4^ cells/well) were first cultured in DMEM medium at 37°C with 10% FBS for 24 h under 5% CO_2_. Then the culture medium was removed, and fresh medium containing 0.1 mg/ml DRF-PEG was added. Following incubation for 0.5 h, the samples were washed with PBS buffer to remove the residual DRF-PEG in the culture medium. Subsequently, cell culture media with different concentrations of Fe^3+^ (0, 5, 20, 100 μM) were added and cultured for another 30 min to allow effective uptake of Fe^3+^. Before imaging, the cells were rinsed with PBS buffer three times to remove the residual Fe^3+^ in the culture medium. CLSM images of the cells were captured at ambient temperature under an excitation wavelength of 488 nm, and the fluorescent signals were collected between 500 and 600 nm.

### Determination of Iron in Fetal Bovine Serum

Fetal bovine serum obtained from Gibco (Germany) was used to investigate the potential application of DRF-PEG in biosystems. To reduce background interference, bovine serum lipids were removed with chloroform–methanol mixture before the test by referring to the reported procedure (Park and Lee, 2020). Stock solutions of diluted fetal bovine serum (10%) with 0.1 mg/ml DRF-PEG were prepared. After adding different concentrations of Fe^3+^ (100–1,000 μM), the fluorescent emission signals of serum samples were recorded.

## Results and discussion

**Scheme 1:**
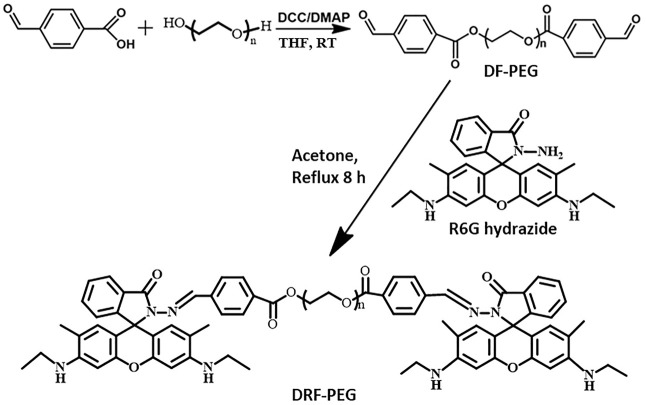
Schematic illustration for the synthesis of di-benzaldehyde terminated poly (ethylene glycol) (DF-PEG) and polymeric sensor containing both PEG chain and rhodamine moiety (DRF-PEG).

**FIGURE 1:**
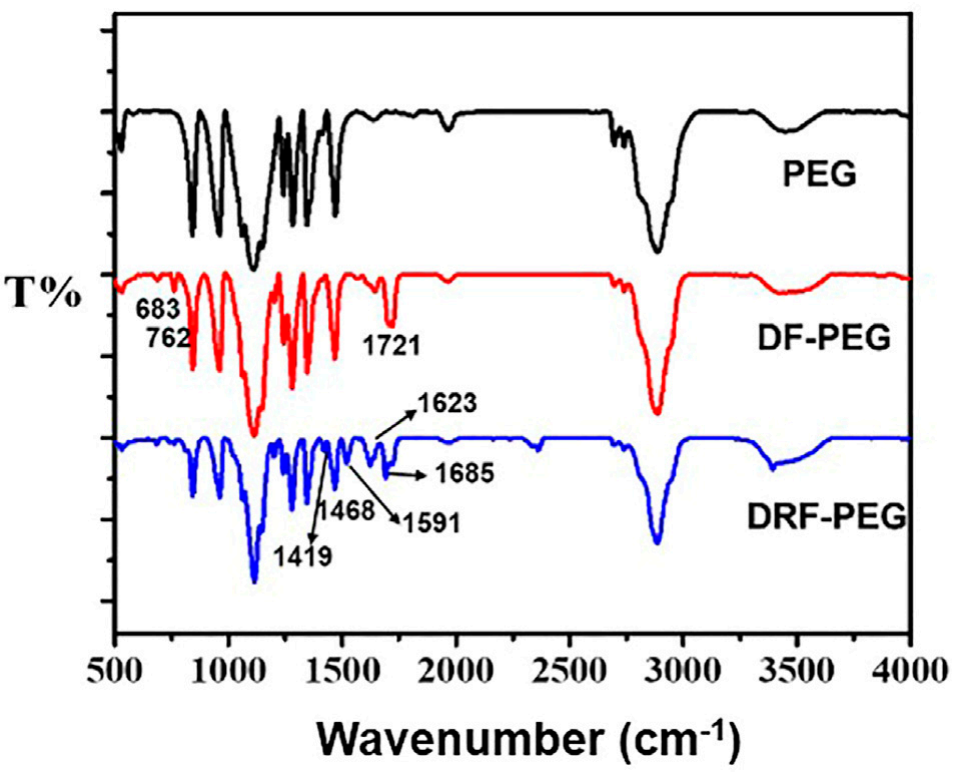
FT-IR spectra of poly (ethylene glycol (PEG), di-benzaldehyde terminated poly (ethylene glycol) (DF-PEG), and polymeric sensor containing both PEG chain and rhodamine moiety (DRF-PEG).

The rhodamine-derived polymeric sensor (DRF-PEG) was facilely synthesized through Schiff base reaction between DF-PEG and Rh with a reaction ratio of 1:2. DF-PEG was synthesized through esterification reaction among poly (ethylene glycol) and 4-carboxybenzaldehyde acid in the presence of DCC/DMAP at ambient temperature. PEG chain in DF-PEG contributes to improve the water solubility of DRF-PEG, while the terminal benzaldehyde group acts as a connecting bridge and will react with amine groups in rhodamine 6G hydrazide. One DF-PEG molecule is covalently bonded with two Rh molecules *via* Schiff base (imine, −N=CH−) linkages. The synthetic route of DRF-PEG is illustrated in Scheme 1. The successful preparation of DRF-PEG was confirmed by FT-IR and ^1^H NMR spectra (Figure 1 and Supplementary Figures S1–S3).

As shown in Figure 1 (red line), the prominent peak located at 1,721 cm^−1^ originated from the stretching vibration of a carbonyl (–C=O) comes from the benzaldehyde groups or the newly formed ester groups in DF-PEG. Compared with the FT-IR spectrum of PEG, newly emerged peaks below 900 cm^−1^ were observed in DF-PEG, which can be ascribed to the characteristic absorption of benzene rings due to the introduction of benzaldehyde groups (Zhang et al., 2011). After combination with rhodamine 6G hydrazide, DRF-PEG with two rhodamine moieties is formed, and characteristic absorption signals of rhodamine moieties (1,419, 1,468, 1,591, and 1,685 cm^−1^) appear on the FT-IR spectrum (blue line). The transmission band at 1,419 cm^−1^ is caused by the C–N deformation of rhodamine moieties, and the peak at 1,519 cm^−1^ can be assigned to the deformation of aromatic rings (Guo et al., 2015; Maity et al., 2018). The sharp peak at 1,623 cm^−1^ suggests the formation of Schiff base linkage (imine, −N=CH−) in DRF-PEG (Jia et al., 2015). The result of FT-IR and ^1^H NMR spectra clearly confirms the successful grafting of rhodamine moieties to DF-PEG.

**FIGURE 2:**
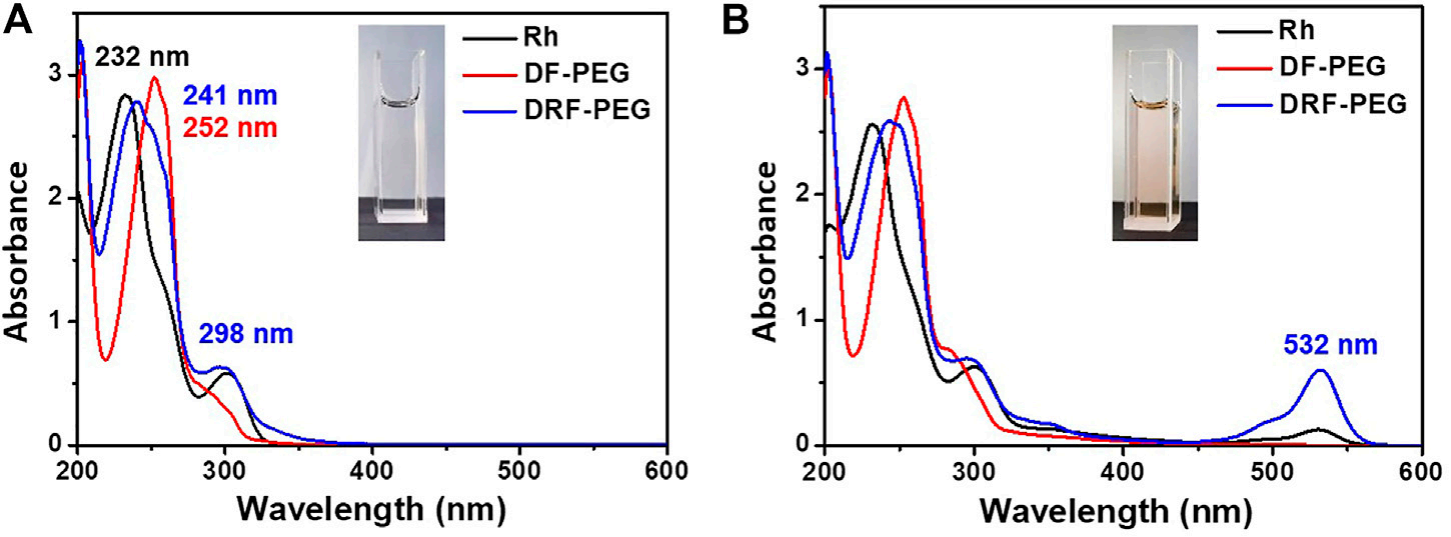
Absorption spectra of rhodamine 6G hydrazide (Rh), DF-PEG, and DRF-PEG **(A)** before and **(B)** after the addition of iron(Fe^3+^).

Figure 2A shows the absorption spectra of Rh, DF-PEG, and DRF-PEG. Because of the poor water solubility of Rh, the absorption experiment herein was conducted in ethanol to ensure the authenticity. DRF-PEG shows two intense absorption bands at 241 and 298 nm, which can be ascribed to intramolecular π–π* and n–π* transition of anthracene moieties in rhodamine units originated from Rh. For DF-PEG, the shoulder peak around 300 nm is weaker than that of Rh and DRF-PEG because there are no anthracene structures in DF-PEG, which possess fewer benzene groups. The intense absorption of DRF-PEG from 250 to 320 nm confirms the successful grafting of rhodamine moieties to DF-PEG (Maity et al., 2018; Dewangan et al., 2019). No absorption above 500 nm is observed for all the solutions in the absence of Fe^3+^. However, with the addition of Fe^3+^, a new absorption peak centered at 532 nm is observed for Rh and DRF-PEG due to the ring opening of spirolactam structure in rhodamine moieties, resulting in the orange color of the solution (Figure 2B). This suggests that the rhodamine units from Rh are successfully conjugated onto DF-PEG, and DRF-PEG can be applied for the recognition of Fe^3+^.

**FIGURE 3:**
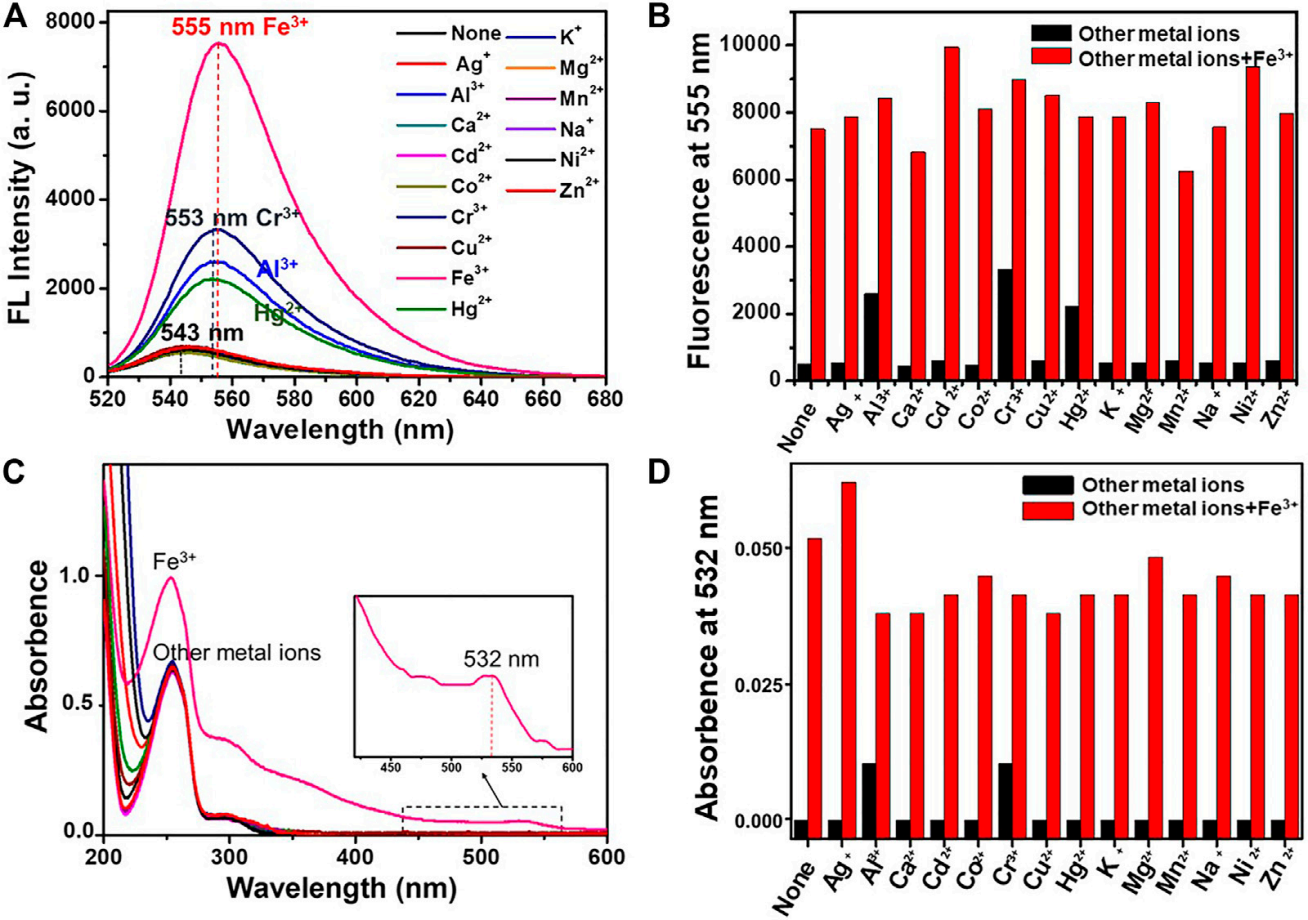
Fluorescence **(A)** and UV-Vis **(C)** spectra of DRF-PEG (0.1 mg/ml) in the presence of various metal ions (10^–4^ M) in water (pH 6.5). Fluorescence emission intensity at 555 nm **(B)** and absorption intensity at 532 nm **(D)** of DRF-PEG (0.1 mg/ml) with various metal ions before and after the addition of Fe^3+^. The black bars are the absorption/fluorescent response of DRF-PEG to various metal ions (10^–4^ M). The red bars are the absorption/fluorescent response after the subsequent addition of Fe^3+^ (10^–4^ M) to the above aqueous solution.

The selectivity of DRF-PEG for the detection of Fe^3+^ in water was investigated by both fluorescence and absorption measurements (Figure 3). After the addition of various metal ions, the spectroscopic measurements were carried out immediately, and the response process was completed instantaneously. As shown in Figures 3A,B, the fluorescence emission intensity of DRF-PEG or DRF-PEG in the presence of most monovalent and divalent metal ions (K^+^, Na^+^, Ag^+^, Mg^2+^, Ca^2+^, Cu^2+^, Co^2+^, Cd^2+^, Zn^2+^, Ni^2+^, and Mn^2+^) was very weak at 543 nm. The addition of Hg^2+^ and Cr^3+^, and Al^3+^ gave rise to a slight increase in fluorescence intensity (4.5- to 6.7-fold) at 553 nm, and a bathochromic shift was observed, whereas the presence of Fe^3+^ induced the most prominent fluorescence enhancement (15-fold) at 555 nm and the largest bathochromic shift (12 nm), suggesting the selective and specific recognition of DRF-PEG toward Fe^3+^. The affinity between Fe^3+^ and DRF-PEG should be the strongest, which has a relationship with the metal-ion radius and ligand configuration (Dey et al., 2017). The chelation of Fe^3+^ induces the structural transformation of rhodamine moiety from spirolactam ring to ring-opened form and brings about fluorescence “turn-ON” effect (Yang et al., 2002). For the selective absorption measurement (Figures 3C,D), an obvious absorption band at 532 nm emerged, and the relative intensity of 253 nm peak was enhanced for DRF-PEG in the presence of Fe^3+^, while for other metal ions, only the band at 253 nm and the shoulder peak at 298 nm were observed. The absorbance at 532 nm by the DRF-PEG-Fe^3+^ complex results in the solution color change from colorless to orange (Supplementary Figure S4). Therefore, DRF-PEG can be invoked as a sensitive colorimetric/fluorescent chemosensor for Fe^3+^ detection in aqueous solutions. It is worth noting that the detection of Fe^3+^ herein by DRF-PEG does not need the assistance of any buffer medium or organic additives, which is much desirable for practical applications.

Anti-interference ability of DRF-PEG to Fe^3+^ is also assessed by fluorescence and UV-Vis absorption spectroscopy at pH 6.5 (Figures 3B, D). Competition experiments are carried out in the presence of Fe^3+^ mixed with equivalent amount of interfering metal ions (K^+^, Na^+^, Ag^+^, Mg^2+^, Ca^2+^, Cu^2+^, Co^2+^, Cd^2+^, Zn^2+^, Hg^2+^, Ni^2+^, Mn^2+^, Cr^3+^, and Al^3+^). Both the fluorescence emission and absorption enhancement of DRF-PEG in the presence of Fe^3+^ were not influenced by all the investigated interfering ions. These results reveal that DRF-PEG shows high selectivity and anti-interference capability for detection of Fe^3+^ over other metal ions. DRF-PEG can serve as a dual-responsive fluorescence turn-on and colorimetric chemosensor for detection of Fe^3+^ with high selectivity in pure aqueous solution. This application of DRF-PEG does not need the assistance of organic solvent, which makes the detection of Fe^3+^ convenient, environmental friendly, and demonstrating great potential in practical environmental and industrial monitoring.

**Scheme 2:**
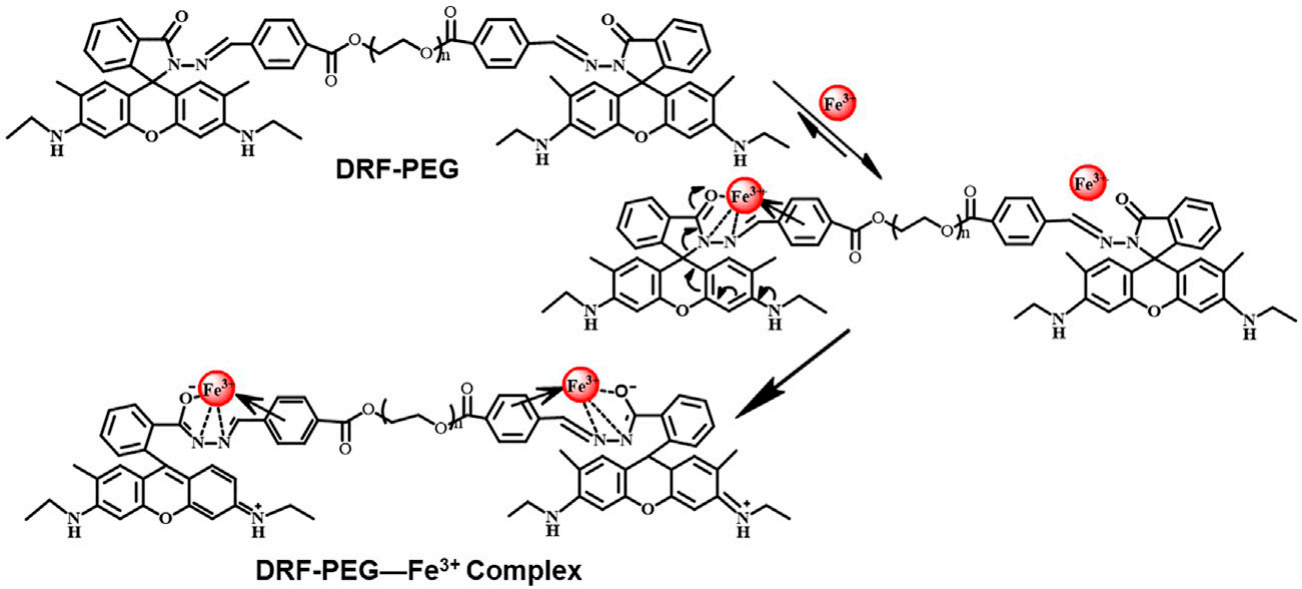
Proposed coordination mechanism of DRF-PEG toward iron (Fe^3+^).

The specific sensing mechanism of DRF-PEG toward Fe^3+^ can be explained by the strong coordination ability of rhodamine moieties toward Fe^3+^ based on our previous reports (Qiu et al., 2013; Qiu et al., 2014; Qiu et al., 2015). As shown in Figure 2, free DRF-PEG is colorless with almost no fluorescence emission. As the nitrogen atoms of Schiff base groups together with the O, N atoms of the five-membered spirolactam ring in DRF-PEG are rich in electrons, they are prone to share electrons with positive metal ions (Yuan et al., 2018). After the addition of Fe^3+^, DRF-PEG starts to coordinate with Fe^3+^, and the coordination will induce intramolecular charge transfer (ICT) in rhodamine moiety, resulting in the ring opening of spirolactam structure and strong fluorescence emission (Scheme 2). As mentioned in Figure 2, the absorption before 320 nm is ascribed to the intramolecular π–π* and n–π* transition in benzene groups. With the increase in Fe^3+^ concentration, the coordination interaction of benzene group and the molecular charge transfer in rhodamine moiety is enhanced. It is supposed the benzene ring from benzaldehyde modified PEG segment in DRF-PEG also participated in the coordination process, and a strong electrostatic interaction is formed between the benzene ring and Fe^3+^. Therefore, the absorption intensity of DRF-PEG before 320 nm increases with Fe^3+^ concentration, which is the reason why DRF-PEG can also act as a colorimetric sensor for Fe^3+^.

**FIGURE 4:**
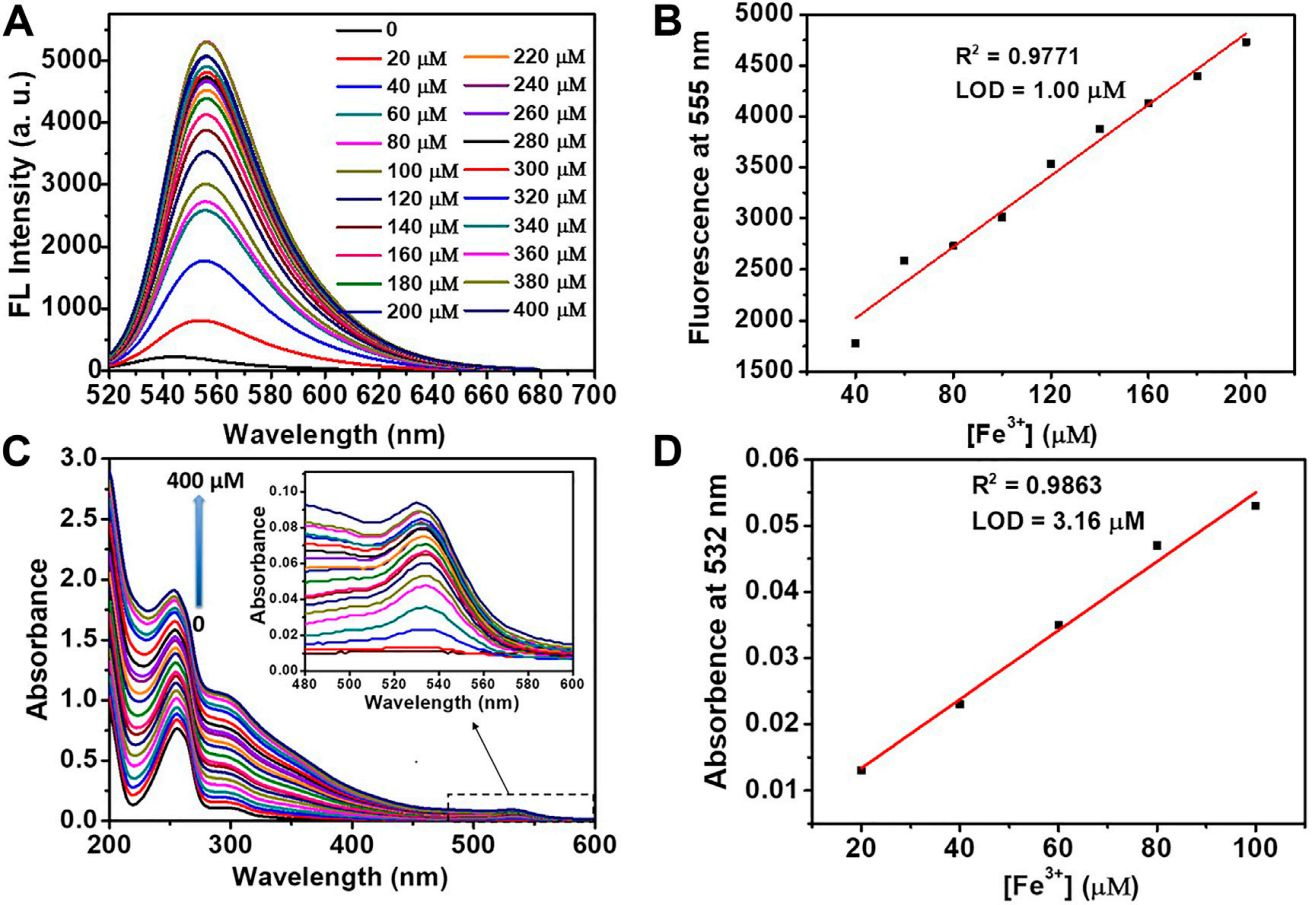
Fluorescence **(A)** and absorption **(C)** titration of DRF-PEG (0.1 mg/ml) with the addition of various concentrations of Fe^3+^ (0–400 μM) in water (pH 6.5). Plot of fluorescent emission intensity at 555 nm **(B)** and UV-Vis absorption intensity at 532 nm **(D)** as a function of [Fe^3+^].

As shown in Figure 4, both the fluorescence intensity (555 nm) and UV-Vis absorbance (532 nm) exhibited a linear relationship with Fe^3+^ concentration for a wide range (0–400 μM). Based on the titration curves, the detection limit of DRF-PEG to Fe^3+^ can be calculated separately from the fluorescence and absorption data through the following equation (Li et al., 2014; Zhou et al., 2017):

Detection limit(DL) = k SD/S

where *k* is the signal-to-noise ratio (*k* = 3), *SD* is the standard deviation of blank DRF-PEG solution, and *S* is the slope of regression line of the titration curve (Zhou et al., 2017). From fluorescence titration data, the limit of detection for DRF-PEG as the fluorescent chemosensor toward Fe^3+^ in water is determined to be 1.00 μM at pH 6.5, which is much lower than the maximum US EPA limit for Fe^3+^ in drinking water (5.4 μM) (Nandre et al., 2014). Similarly, the *DL* value of DRF-PEG as a colorimetric sensor in water for Fe^3+^ at pH 6.5 is estimated to be 3.16 μM based on the absorbance titration results.

**FIGURE 5:**
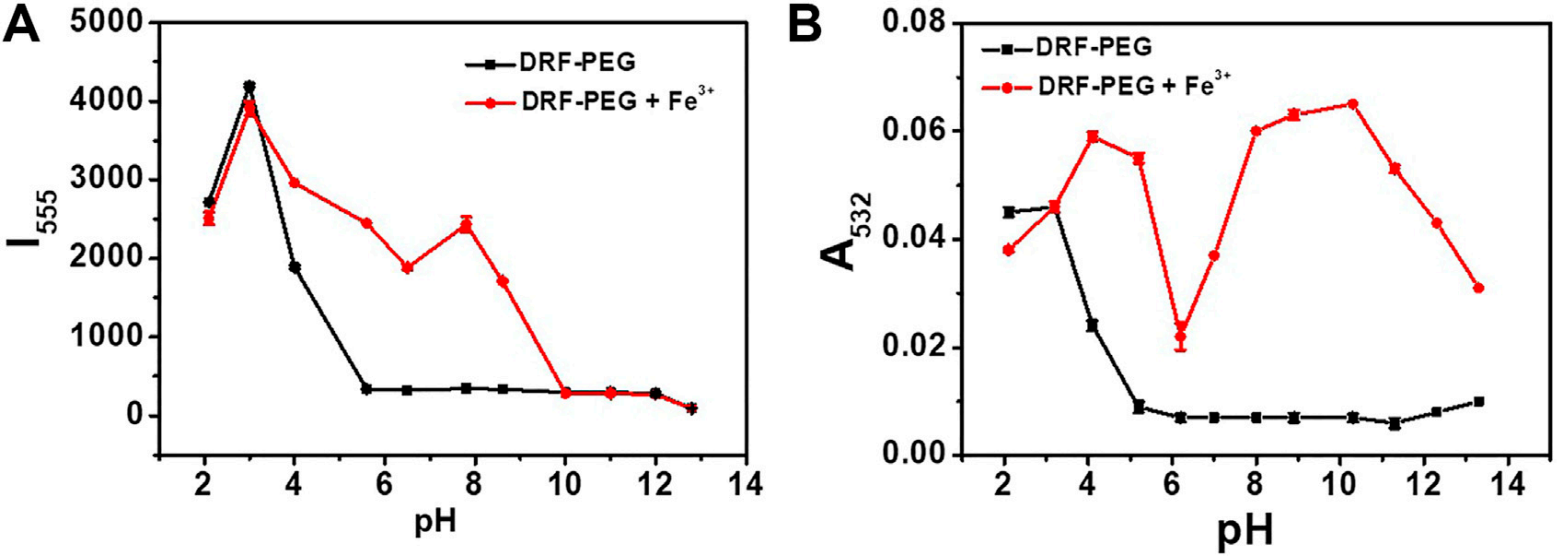
**(A)** Influence of pH on the fluorescent emission intensity of DRF-PEG (0.1 mg/ml) at 555 nm before and after addition of Fe^3+^ in water. **(B)** Influence of pH on the absorption intensity of DRF (0.1 mg/ml) at 532 nm before and after addition of Fe^3+^ ions in water.

For the efficient detection of metal ions in practical applications, sensors should possess the ability to be operated in a broad pH range. Namely, the fluorescent/absorption response of the sensor should take place and not be affected by the solution pH (Li et al., 2014; Li et al., 2015). The effect of pH on the absorption and fluorescent emission of DRF-PEG to Fe^3+^ is checked in the pH range of 2.0–13.3 (Figure 5). Hydrogen ions can also induce ring opening of the spirolactam structure in rhodamine moiety (Song Y. et al., 2019; Jiao et al., 2018). At pH 3–5, free DRF-PEG exhibits strong fluorescence/absorption due to the existence of abundant hydrogen ions in the solution. However, the marked increase in the fluorescence/absorption intensity after the addition of Fe^3+^ suggests that DRF-PEG can be applied for the recognition of Fe^3+^ in this pH range. It is proposed that the existence of H^+^ will restrain the coordination of Fe^3+^ with DRF-PEG. The fluorescence/absorption intensity first decreased with the increase in pH (5 < pH < 7), then increased when the amount of H^+^ is very few because the interference from hydrogen ions disappeared (7 < pH < 10). At pH >10, a large amount of Fe^3+^ would be precipitated by OH^−^ according to the solubility product rule, so the fluorescence/absorption intensity of DRF-PEG decreases dramatically since pH 10. As shown in Figure 5, DRF-PEG can be used as a fluorescent chemosensor for Fe^3+^ in the pH range of 3.0–10.0 and served as a colorimetric sensor for Fe^3+^ in the pH range of 3.2–13.3. Therefore, DRF-PEG has great tolerance to the solution pH, suggesting the diverse applications of DRF-PEG in metal ion detection.

**FIGURE 6:**
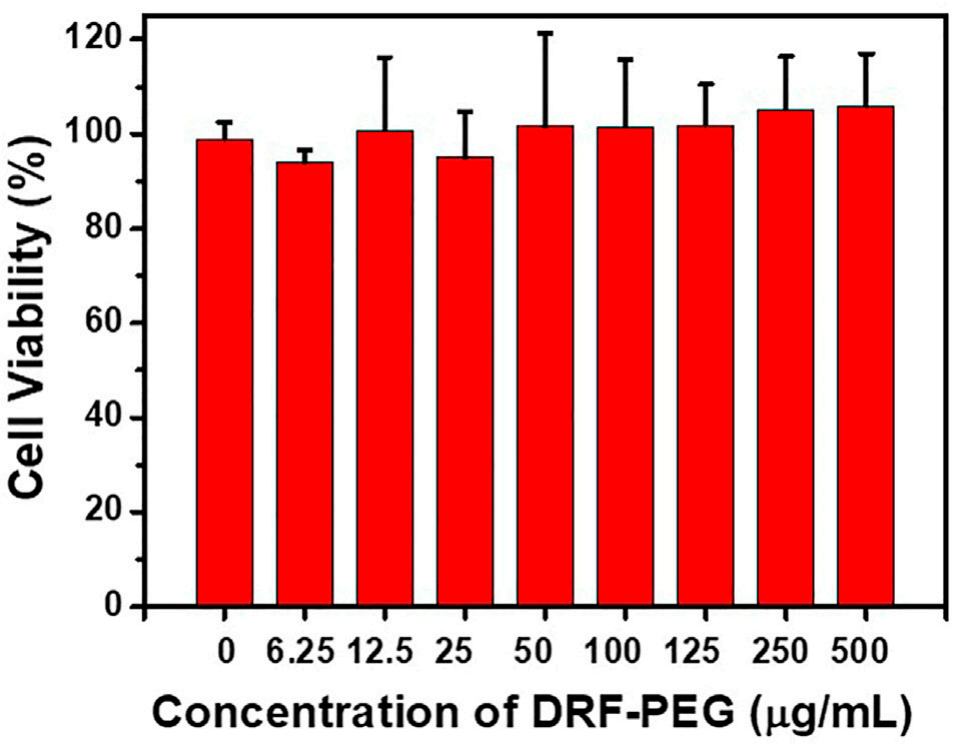
Cell viability values of human cervical carcinoma cells (HeLa) vs. the concentration of DRF-PEG. HeLa cells were incubated with different concentrations of DRF-PEG (0–500 μg/ml) for 24 h.

Biocompatibility and cytotoxicity are important criteria to assess the potential application of chemical sensors in biological and medical studies (Shi et al., 2016; Dong et al., 2017). The cytotoxicity of DRF-PEG (0–500 μg/ml) to HeLa cells was evaluated through MTT assays. As shown in Figure 6, the cell viability for all the tested samples is higher than 95% even under a high concentration of DRF-PEG at 500 μg/ml after incubation for 24 h. The high cell viability of HeLa cell indicates the high biocompatibility and low cytotoxicity of DRF-PEG, suggesting high potential in living cell imaging or *in vitro* tests.

**FIGURE 7:**
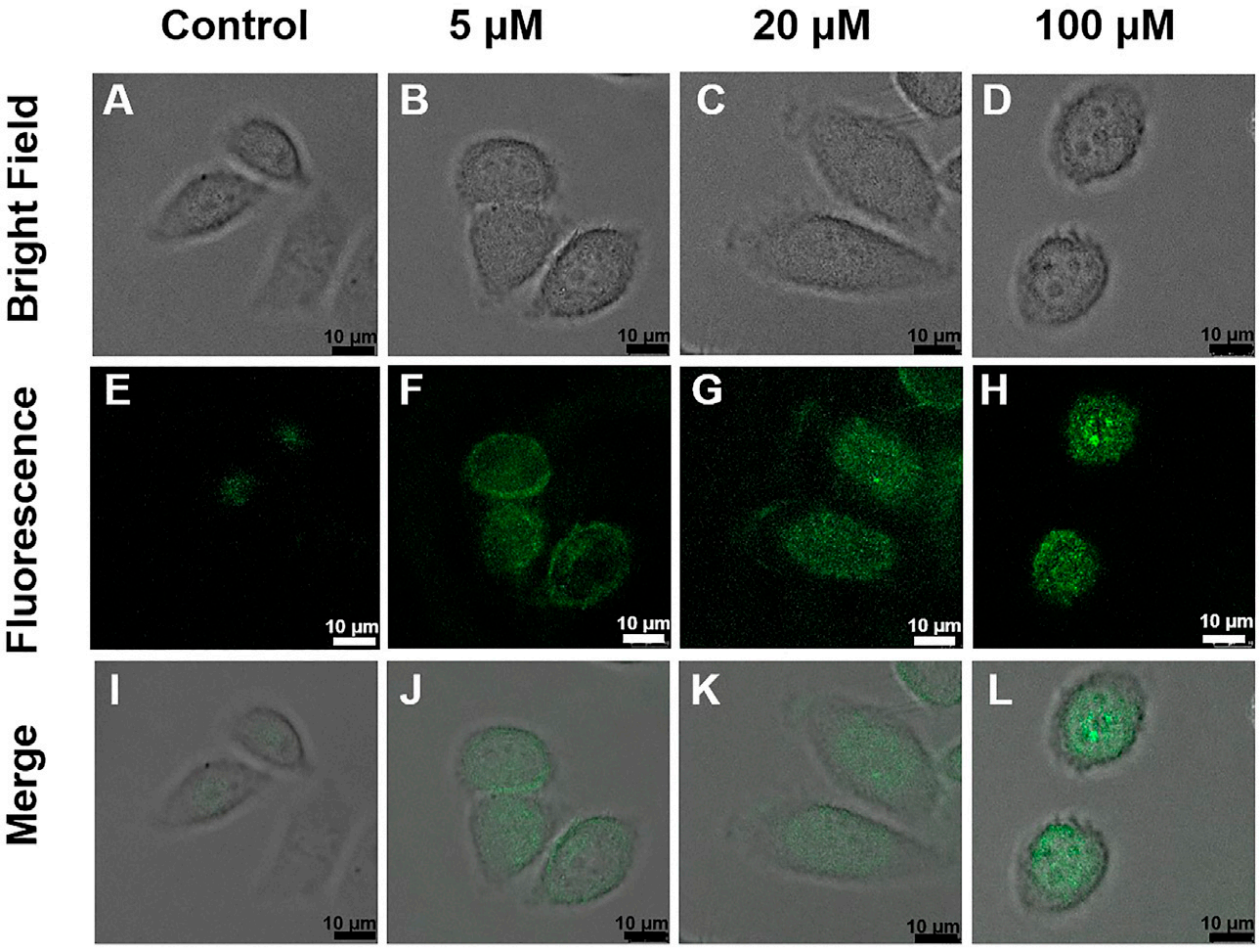
Confocal laser scanning microscopy (CLSM) images of HeLa cells treated with DRF-PEG (0.1 mg/ml) and different concentrations of Fe^3+^ (0, 5, 20, and 100 μM). The upper panel: bright field images, the middle: dark field, and the lower: overlays of the bright and dark field images. HeLa cells were first incubated with DRF-PEG (0.1 mg/ml) for 30 min and then with different concentrations of Fe^3+^ as 0 μM **(A, E, and I)**, 5 μM **(B, F, and J)**, 20 μM **(C, G, and K)**, and 100 μM **(D, H, and L)** for another 30 min at 37°C.

Encouraged by the biocompatibility and high sensing performance of DRF-PEG, intracellular detection of Fe^3+^ and bio-imaging capability of DRF-PEG are tested in living HeLa cells by CLSM. HeLa cells were first incubated with 0.1 mg/ml DRF-PEG for 30 min, washed by PBS buffer, and then cultured with different concentrations of Fe^3+^ for another 30 min. As shown in Figure 7, controlled cells without additional supplement of Fe^3+^ display faint fluorescence emission because of a trace amount of free iron or non-transferrin-bound ions in the culture medium, while bright and strong green fluorescence is observed for HeLa cells incubated with extra Fe^3+^ from 5 to 100 μM. The fluorescent signals are specifically located in the intracellular areas suggesting that DRF-PEG possesses good cell membrane permeability and can be used for the monitoring of Fe^3+^ in living cells without any transfection agents. Intracellular fluorescence emission intensity of HeLa cells is enhanced with the amount of Fe^3+^ added (Figure 7 and Supplementary Figure S5). When the concentration of Fe^3+^ is low (5 μM), the fluorescence is mainly concentrated in the cytoplasm area of HeLa cells. With the increase in Fe^3+^, intracellular uptake of Fe^3+^ increases, and fluorescent signals start to spread over the whole cell area. Obviously, HeLa cells incubated with 100 μM of Fe^3+^ exhibit the strongest fluorescent emission after the same incubation period (30 min) with bright green fluorescence in the nucleus. According to the distribution of fluorescence signals, DRF-PEG can be implemented to monitor the intracellular absorption path of Fe^3+^, showing enormous potential in intracellular imaging and visually monitor Fe^3+^ in living systems.

**FIGURE 8:**
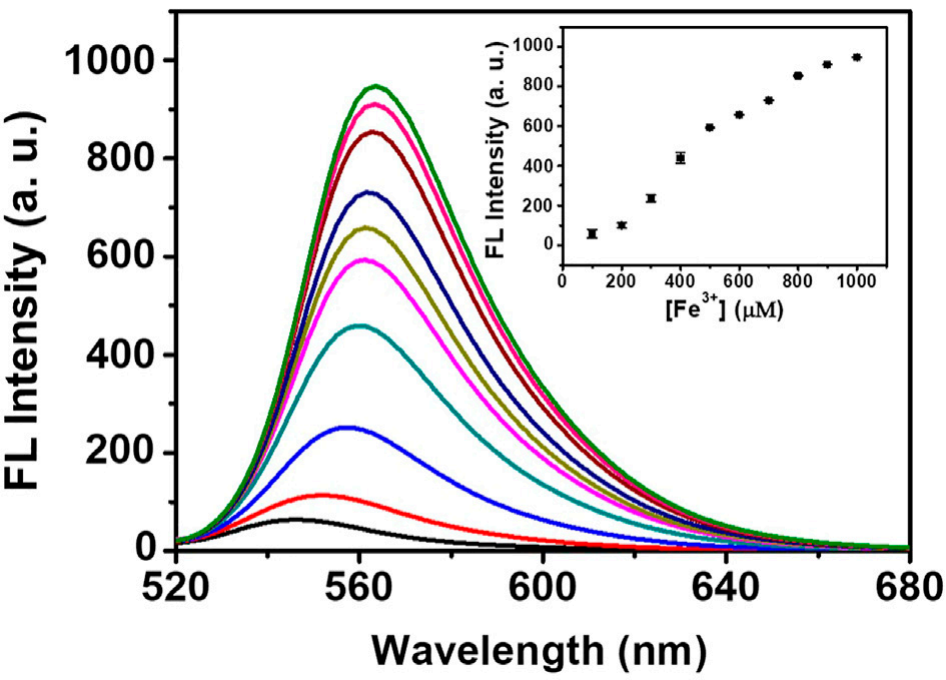
Fluorescence spectra of DRF-PEG in diluted serum (10%) with different concentrations of Fe^3+^ (100–1,000 μM). The inset is the fluorescence intensity as a function of [Fe^3+^].

As a proof of concept, the detection performance of DRF-PEG toward Fe^3+^ in fetal bovine serum is examined. As shown in Figure 8, the fluorescence emission intensity of DRF-PEG in 10% bovine serum shows an increasing trend with the concentration of additive Fe^3+^ in the range of 100–1,000 μM, and the minimum responsive concentration of Fe^3+^ is 100 μM. The biosensing performance of DRF-PEG toward Fe^3+^ can be ascribed to the anti-fouling properties of PEG chains from benzaldehyde-modified PEG segments. Results showed that the DRF-PEG possesses sensing capability toward Fe^3+^ in biological samples, implying its potential applications to serve as a bio-sensor in biomedical fields. In comparison with the recent rhodamine-based chemosensors for Fe^3+^ (Table 1), DRF-PEG shows excellent detection performance in pure aqueous solution with potential biological applications.

**TABLE 1:** Comparison of polymeric sensor containing both PEG chain and rhodamine moiety (DRF-PEG) with other rhodamine-based chemosensors for iron (Fe^3+^).

Sensor	Solvent	DL (μM)	pH range	References
Probe M3	Acetonitrile/Tris-HCl (3:7, v/v)	5.2	4.2–8.8	Zhang M. et al. (2020)
Rh-AQ	CH_3_CN/HEPES (50%)	3.5	NA	Huang et al. (2014)
RhBNC	THF	0.16	NA	Vijay et al. (2019)
Probe 1	MeOH/H_2_O (1/1, v/v)	3.76	NA	Liu et al. (2020)
RBPO	EtOH/H_2_O (3:1, v/v)	0.067	NA	Song F. et al. (2019)
RDG2	Water	2.09	4.0–7.0	Chen and Fang (2018)
RL	DMSO/H_2_O(1：1, v/v)	0.28	4.0–11.0	Zhou et al. (2017)
DRF-PEG	Water	1.0	3.2–13.3	This work

DL, detection limit, NA, not available.

## Conclusion

In summary, a water-soluble dual colorimetric/fluorescent responsive chemosensor (DRF-PEG) for the detection of Fe^3+^ with good biocompatibility was constructed by combining the specific binding effect of rhodamine moiety and the excellent water solubility of difunctionalized PEG. Based on the strong coordination of N and O atoms from rhodamine moieties and benzene groups from DF-PEG with Fe^3+^, DRF-PEG can be used for colorimetric/fluorescent sensing of Fe^3+^ in pure aqueous solutions without any aid of organic solvent or buffer medium in a wide pH range. DRF-PEG possesses excellent biocompatibility for living cell imaging and the intracellular detection of Fe^3+^ in HeLa cells. By virtue of the anti-fouling properties of PEG groups, DRF-PEG shows great potential in detecting iron ions in complex biological samples, which is promising in biological imaging and medical diagnosis.

## Data Availability

The original contributions presented in the study are included in the article/Supplementary Material, further inquiries can be directed to the corresponding author.

## References

[B1] Cao J. Ding L. Hu W. Chen X. Chen X. Fang Y. (2014). Ternary System Based on Fluorophore-Surfactant Assemblies-Cu2+ for Highly Sensitive and Selective Detection of Arginine in Aqueous Solution. Langmuir 30 (50), 15364–15372. 10.1021/la5039798 25453500

[B2] Carter K. P. Young A. M. Palmer A. E. (2014). Fluorescent Sensors for Measuring Metal Ions in Living Systems. Chem. Rev. 114 (8), 4564–4601. 10.1021/cr400546e 24588137 PMC4096685

[B3] Chen Q. Fang Z. (2018). Two Sugar-Rhodamine "Turn-On" Fluorescent Probes for the Selective Detection of Fe^3+^ . Spectrochim. Acta A: Mol. Biomol. Spectrosc. 193, 226–234. 10.1016/j.saa.2017.12.023 29247919

[B4] Choudhury N. Ruidas B. Mukhopadhyay C. D. De P. (2020). Rhodamine-Appended Polymeric Probe: An Efficient Colorimetric and Fluorometric Sensing Platform for Hg2+ in Aqueous Medium and Living Cells. ACS Appl. Polym. Mater. 2, 5077–5085. 10.1021/acsapm.0c00878

[B5] Dewangan S. Barik T. Parida R. Mawatwal S. Dhiman R. Giri S. (2019). Solvent Free Synthesis of Ferrocene Based Rhodamine - Hydrazone Molecular Probe with Improved Bioaccumulation for Sensing and Imaging Applications. J. Organomet. Chem. 904, 120999. 10.1016/j.jorganchem.2019.120999

[B6] Dey S. Sarkar S. Maity D. Roy P. (2017). Rhodamine Based Chemosensor for Trivalent Cations: Synthesis, Spectral Properties, Secondary Complex as Sensor for Arsenate and Molecular Logic gates. Sens. Actuators B: Chem. 246, 518–534. 10.1016/j.snb.2017.02.094

[B7] Dong J. X. Wang Z. L. Yang Y. Gao Z. F. Li B. L. Jiang H. H. (2017). Bio-friendly Maillard Reaction Fluorescent Products from Glutathione and Ascorbic Acid for the Rapid and Label-free Detection of Fe^3+^ in Living Cells. J. Mater. Chem. B 5 (4), 707–713. 10.1039/c6tb02449a 32263838

[B8] Fan K. Wang X. Yu S. Han G. Xu D. Zhou L. (2019). A Chitosan-Based Fluorescent Hydrogel for Selective Detection of Fe2+ Ions in Gel-To-Sol Mode and Turn-Off Fluorescence Mode. Polym. Chem. 10 (37), 5037–5043. 10.1039/c9py01179j

[B9] Frankowski M. Zioła-Frankowska A. Siepak J. (2010). New Method for Speciation Analysis of Aluminium Fluoride Complexes by HPLC-FAAS Hyphenated Technique. Talanta 80 (5), 2120–2126. 10.1016/j.talanta.2009.11.018 20152461

[B10] Geng T.-M. Zhang W.-Y. Li D.-K. Xia H.-Y. Wang Y. Wang Z.-Q. (2017). The Chromogenic and Fluorescent Sensing Properties for a Water Soluble Polymeric Chemosensor Bearing Rhodamine Ethanediamine Moieties with Oxethyl (OCH 2 CH 2 ) as a Spacer. J. Environ. Chem. Eng. 5 (1), 906–914. 10.1016/j.jece.2017.01.017

[B11] Guo R. Zhou S. Li Y. Li X. Fan L. Voelcker N. H. (2015). Rhodamine-Functionalized Graphene Quantum Dots for Detection of Fe3+ in Cancer Stem Cells. ACS Appl. Mater. Inter. 7 (43), 23958–23966. 10.1021/acsami.5b06523 26317667

[B12] Hou J.-T. Ren W. X. Li K. Seo J. Sharma A. Yu X.-Q. (2017). Fluorescent Bioimaging of pH: from Design to Applications. Chem. Soc. Rev. 46 (8), 2076–2090. 10.1039/c6cs00719h 28317979

[B13] Huang J. Xu Y. Qian X. (2014). Rhodamine-based Fluorescent Off-On Sensor for Fe3+ - in Aqueous Solution and in Living Cells: 8-aminoquinoline Receptor and 2 : 1 Binding. Dalton Trans. 43 (16), 5983–5989. 10.1039/c3dt53159g 24442254

[B14] Jia H. Li Z. Wang X. Zheng Z. (2015). Facile Functionalization of a Tetrahedron-like PEG Macromonomer-Based Fluorescent Hydrogel with High Strength and its Heavy Metal Ion Detection. J. Mater. Chem. A. 3 (3), 1158–1163. 10.1039/c4ta05736h

[B15] Jia X. Gong D. Zhao J. Ren H. Wang J. Zhang X. (2018). Zwitterion-functionalized Polymer Microspheres as a Sorbent for Solid Phase Extraction of Trace Levels of V(V), Cr(III), As(III), Sn(IV), Sb(III) and Hg(II) Prior to Their Determination by ICP-MS. Microchim. Acta 185 (4), 228. 10.1007/s00604-018-2766-x 29594828

[B16] Jiao Y. Zhou L. He H. Yin J. Gao Q. Wei J. (2018). A Novel Rhodamine B-Based "Off-On'' Fluorescent Sensor for Selective Recognition of Copper (II) Ions. Talanta 184, 143–148. 10.1016/j.talanta.2018.01.073 29674025

[B17] Jin Q. Chen S. Jiang H. Wang Y. Zhang L. Liu M. (2018). Self-Assembly of Amphiphilic Schiff Base and Selectively Turn on Circularly Polarized Luminescence by Al^3+^ . Langmuir 34 (47), 14402–14409. 10.1021/acs.langmuir.8b03019 30398358

[B18] Jung Y. Ju I. G. Choe Y. H. Kim Y. Park S. Hyun Y.-M. (2019). Hydrazine Exposé: The Next-Generation Fluorescent Probe. ACS Sens. 4 (2), 441–449. 10.1021/acssensors.8b01429 30652852

[B19] Li C.-Y. Zou C.-X. Li Y.-F. Tang J.-L. Weng C. (2014). A New Rhodamine-Based Fluorescent Chemosensor for Fe^3+^ and its Application in Living Cell Imaging. Dyes Pigm. 104, 110–115. 10.1016/j.dyepig.2014.01.003

[B20] Li G. Tao F. Wang H. Li Y. Wang L. (2015). A Novel Reversible Colorimetric Chemosensor for Rapid Naked-Eye Detection of Cu^2+^ in Pure Aqueous Solution. Sens. Actuators B: Chem. 211, 325–331. 10.1016/j.snb.2015.01.105

[B21] Li G. Tao F. Liu Q. Wang L. Wei Z. Zhu F. (2016). A Highly Selective and Reversible Water-Soluble Polymer Based-Colorimetric Chemosensor for Rapid Detection of Cu^2+^ in Pure Aqueous Solution. New J. Chem. 40 (5), 4513–4518. 10.1039/c5nj03526k

[B22] Li G. Bai L. Tao F. Deng A. Wang L. (2018). A Dual Chemosensor for Cu^2+^ and Hg^2+^ Based on a Rhodamine-Terminated Water-Soluble Polymer in 100% Aqueous Solution. Analyst 143 (22), 5395–5403. 10.1039/c8an01130c 30295689

[B23] Lin M.-H. Ren X.-X. Ning X.-M. Liu D.-Y. Qian J. (2020). Improving Ion Selectivity of 1,4,7-Triazacyclononane-Based Receptor by Zinc Coordination: "Turn-On" Chemosensor for Br- and Fe3+ Ions. Langmuir 36 (44), 13218–13226. 10.1021/acs.langmuir.0c02072 33104351

[B24] Liu X. Miller II A. L. II Yaszemski M. J. Lu L. (2015). Biodegradable and Crosslinkable PPF-PLGA-PEG Self-Assembled Nanoparticles Dual-Decorated with Folic Acid Ligands and Rhodamine B Fluorescent Probes for Targeted Cancer Imaging. RSC Adv. 5 (42), 33275–33282. 10.1039/c5ra04096e PMC8942413 35330847

[B25] Liu Z. Purro M. Qiao J. Xiong M. P. (2017). Multifunctional Polymeric Micelles for Combining Chelation and Detection of Iron in Living Cells. Adv. Healthc. Mater. 6 (17), 1700162. 10.1002/adhm.201700162 PMC5587393 28661064

[B26] Liu Y. Zhao C. Zhao X. Liu H. Wang Y. Du Y. (2020). A Selective N,N-dithenoyl-rhodamine Based Fluorescent Probe for Fe3+ Detection in Aqueous and Living Cells. J. Environ. Sci. 90, 180–188. 10.1016/j.jes.2019.12.005 32081314

[B27] Maity S. Parshi N. Prodhan C. Chaudhuri K. Ganguly J. (2018). Characterization of a Fluorescent Hydrogel Synthesized Using Chitosan, Polyvinyl Alcohol and 9-anthraldehyde for the Selective Detection and Discrimination of Trace Fe3+ and Fe2+ in Water for Live-Cell Imaging. Carbohydr. Polym. 193, 119–128. 10.1016/j.carbpol.2018.03.073 29773363

[B28] Nandre J. Patil S. Patil V. Yu F. Chen L. Sahoo S. (2014). A Novel Fluorescent "Turn-On" Chemosensor for Nanomolar Detection of Fe(III) from Aqueous Solution and its Application in Living Cells Imaging. Biosens. Bioelectron. 61, 612–617. 10.1016/j.bios.2014.06.017 24967750

[B29] Nayab P. S. Shkir M. (2017). A Dual Responsive Colorimetric and Fluorescent Reversible Turn-On Chemosensor for Iron (Fe^3+^): Computational and Spectroscopic Investigations. Sens. Actuators B: Chem. 245, 395–405. 10.1016/j.snb.2017.01.072

[B30] Park T. E. Lee S. H. (2020). A Micellized Fluorescence Sensor Based on Amplified Quenching for Highly Sensitive Detection of Non-transferrin-bound Iron in Serum. Dalton Trans. 49 (15), 4660–4664. 10.1039/d0dt00026d 32115591

[B31] Qiu X. Han S. Gao M. (2013). Highly Sensitive and Selective Detection of Cu(ii) by Periodic Mesoporous Rhodamine Derivative-Based Organosilicas with crystal-like Pore walls. J. Mater. Chem. A. 1 (4), 1319–1325. 10.1039/c2ta00411a

[B32] Qiu X. Han S. Hu Y. Gao M. Wang H. (2014). Periodic Mesoporous Organosilicas for Ultra-high Selective Copper(ii) Detection and Sensing Mechanism. J. Mater. Chem. A. 2 (5), 1493–1501. 10.1039/c3ta14314g

[B33] Qiu X. Han S. Hu Y. Sun B. (2015). Ratiometric Fluorescent Nanosensors for Copper(II) Based on Bis(rhodamine)-Derived PMOs with J-type Aggregates. Chem. Eur. J. 21 (10), 4126–4132. 10.1002/chem.201406143 25640601

[B34] Rong G. Corrie S. R. Clark H. A. (2017). *In Vivo* biosensing: Progress and Perspectives. ACS Sens. 2 (3), 327–338. 10.1021/acssensors.6b00834 28723197 PMC5520645

[B35] Shi B. Su Y. Zhang L. Huang M. Liu R. Zhao S. (2016). Nitrogen and Phosphorus Co-doped Carbon Nanodots as a Novel Fluorescent Probe for Highly Sensitive Detection of Fe3+ in Human Serum and Living Cells. ACS Appl. Mater. Inter. 8 (17), 10717–10725. 10.1021/acsami.6b01325 27014959

[B36] Song Y. Ma Z. Fang H. Zhang Q. Zhou Q. Chen Z. (2020). Au Sputtered Paper Chromatography Tandem Raman Platform for Sensitive Detection of Heavy Metal Ions. ACS Sens. 5 (5), 1455–1464. 10.1021/acssensors.0c00395 32349471

[B37] Song F. Yang C. Liu H. Gao Z. Zhu J. Bao X. (2019). Dual-binding Pyridine and Rhodamine B Conjugate Derivatives as Fluorescent Chemosensors for Ferric Ions in Aqueous media and Living Cells. Analyst 144 (9), 3094–3102. 10.1039/c8an01915k 30920566

[B38] Song Y. Zheng Y. Zhang S. Song Y. Niu M. Li Y. (2019). Always-on and Water-Soluble Rhodamine Amide Designed by Positive Charge Effect and Application in Mitochondrion-Targetable Imaging of Living Cells. Sens. Actuators B: Chem. 286, 32–38. 10.1016/j.snb.2019.01.107

[B39] Vijay N. Wu S. P. Velmathi S. (2019). Turn on Fluorescent Chemosensor Containing Rhodamine B Fluorophore for Selective Sensing and *In Vivo* Fluorescent Imaging of Fe3+ Ions in HeLa Cell Line and Zebrafish. J. Photochem. Photobiol. A: Chem. 384, 112060. 10.1016/j.jphotochem.2019.112060

[B40] Wu W. Chen A. Tong L. Qing Z. Langone K. P. Bernier W. E. (2017). Facile Synthesis of Fluorescent Conjugated Polyelectrolytes Using Polydentate Sulfonate as Highly Selective and Sensitive Copper(II) Sensors. ACS Sens. 2 (9), 1337–1344. 10.1021/acssensors.7b00400 28795572

[B41] Wu M. Suo F. Zhou J. Gong Q. Bai L. Chen B. (2018). Paper-based Fluorogenic Device for Detection of Copper Ions in a Biological System. ACS Appl. Bio Mater. 1, 1523–1529. 10.1021/acsabm.8b00435 34996203

[B42] Yan B. Huang J. Han L. Gong L. Li L. Israelachvili J. N. (2017). Duplicating Dynamic Strain-Stiffening Behavior and Nanomechanics of Biological Tissues in a Synthetic Self-Healing Flexible Network Hydrogel. ACS Nano 11 (11), 11074–11081. 10.1021/acsnano.7b05109 28956900

[B43] Yang X.-F. Guo X.-Q. Zhao Y.-B. (2002). Development of a Novel Rhodamine-type Fluorescent Probe to Determine Peroxynitrite. Talanta 57 (5), 883–890. 10.1016/s0039-9140(02)00120-0 18968692

[B44] Yuan J. Wu S.-Q. Liu M.-J. Sato O. Kou H.-Z. (2018). Rhodamine 6G-Labeled Pyridyl Aroylhydrazone Fe(II) Complex Exhibiting Synergetic Spin Crossover and Fluorescence. J. Am. Chem. Soc. 140 (30), 9426–9433. 10.1021/jacs.8b00103 29983062

[B45] Zhang Y. Tao L. Li S. Wei Y. (2011). Synthesis of Multiresponsive and Dynamic Chitosan-Based Hydrogels for Controlled Release of Bioactive Molecules. Biomacromolecules 12 (8), 2894–2901. 10.1021/bm200423f 21699141

[B46] Zhang D. Yao Y. Wu J. Protsak I. Lu W. He X. (2019). Super Hydrophilic Semi-IPN Fluorescent Poly(N-(2-hydroxyethyl)acrylamide) Hydrogel for Ultrafast, Selective, and Long-Term Effective Mercury(II) Detection in a Bacteria-Laden System. ACS Appl. Bio Mater. 2 (2), 906–915. 10.1021/acsabm.8b00761 35016294

[B47] Zhang M. Shen C. Jia T. Qiu J. Zhu H. Gao Y. (2020). One-step Synthesis of Rhodamine-Based Fe3+ Fluorescent Probes via Mannich Reaction and its Application in Living Cell Imaging. Spectrochim. Acta Part A: Mol. Biomol. Spectrosc. 231, 118105. 10.1016/j.saa.2020.118105 32006914

[B48] Zhang Y. Gutiérrez M. Chaudhari A. K. Tan J.-C. (2020). Dye-Encapsulated Zeolitic Imidazolate Framework (ZIF-71) for Fluorochromic Sensing of Pressure, Temperature, and Volatile Solvents. ACS Appl. Mater. Inter. 12, 37477–37488. 10.1021/acsami.0c10257 32700893

[B49] Zhou F. Leng T.-H. Liu Y.-J. Wang C.-Y. Shi P. Zhu W.-H. (2017). Water-soluble Rhodamine-Based Chemosensor for Fe 3+ with High Sensitivity, Selectivity and Anti-interference Capacity and its Imaging Application in Living Cells. Dyes Pigm. 142, 429–436. 10.1016/j.dyepig.2017.03.057

