# Supplementary material for: Facile Synthesis of Water-Soluble Rhodamine-Based Polymeric Chemosensors via Schiff Base Reaction for Fe3+ Detection and Living Cell Imaging
Source: Front Chem. 2022 Feb 28;10:845627. doi: 10.3389/fchem.2022.845627 (PMC8919081; doi:10.3389/fchem.2022.845627)
Supplement: Supplementary file 1 [file DataSheet1.docx]

Supporting Information

Facile Synthesis of Water-Soluble Rhodamine-Based Polymeric Chemosensors via Schiff Base Reaction for Fe^3+^ Detection and Living Cell Imaging

**Xiaoyong Qiu^1^, Jun Huang^2^, Ning Wang^1^, Kaijie Zhao^1^, Jiwei Cui^1^, Jingcheng Hao^1*^**

^1^Key Laboratory of Colloid and Interface Chemistry of the Ministry of Education, School of Chemistry and Chemical Engineering, Shandong University, Jinan 250100, China

^2^ Center for Advanced Jet Engineering Technologies (CaJET), Key Laboratory of High Efficiency and Clean Mechanical Manufacture of the Ministry of Education, School of Mechanical Engineering, Shandong University, Jinan, Shandong, 250061, China

* Corresponding author. Tel.: +86-531-88366074. E-mail: jhao@sdu.edu.cn

**1. Synthesis of Rhodamine 6G Hydrazide**

Rhodamine 6G was synthesized through the reported method (Yang et al., 2002) Rhodamine 6G (0.958 g, 2 mmol) was firstly dissolved in 30 mL methanol, then hydrazine monohydrate (5 mL, 50%) in 20 mL methanol was added drop by drop under heating. The mixture was refluxed for 8 h and cooled overnight. Then the mixture was filtered and washed with ethanol/water for three times. Rhodamine 6G hydrazide was obtained as a pink solid with a yield of 80%. ^1^H NMR (400 MHz, DMSO-*d_6_*, δ): 1.21 (t, J = 7.1 Hz, 6H; CH_3_), 1.87 (s, 6H; CH_3_), 3.10-3.16 (m, 4H; NCH_2_), 4.21 (s, 2H; NH_2_), 4.99 (t, J =5.44 Hz, 2H; NH), 6.10 (s, 2H; ArH), 6.27 (s, 2H; ArH), 6.92-6.94 (m, 1H; CH), 7.44-7.48 (m, 2H; ArH), 7.74-7.77 (m, 1H; ArH).

**2. Extraction of Lipid from Fetal Bovine Serum**

The procedure for the extraction of lipid from serum is conducted according to the reported literature (Park et al., 2020). Fetal bovine serum (5 mL) is thoroughly mixed with chloroform-methanol (*v: v* = 2:1). The solution is centrifuged at 5000 rpm for 5 min, lipids are separated and colorless liquid is obtained for the subsequent experiment.

**3. ^1^H NMR Spectra of Rh, DF-PEG, DRF-PEG**


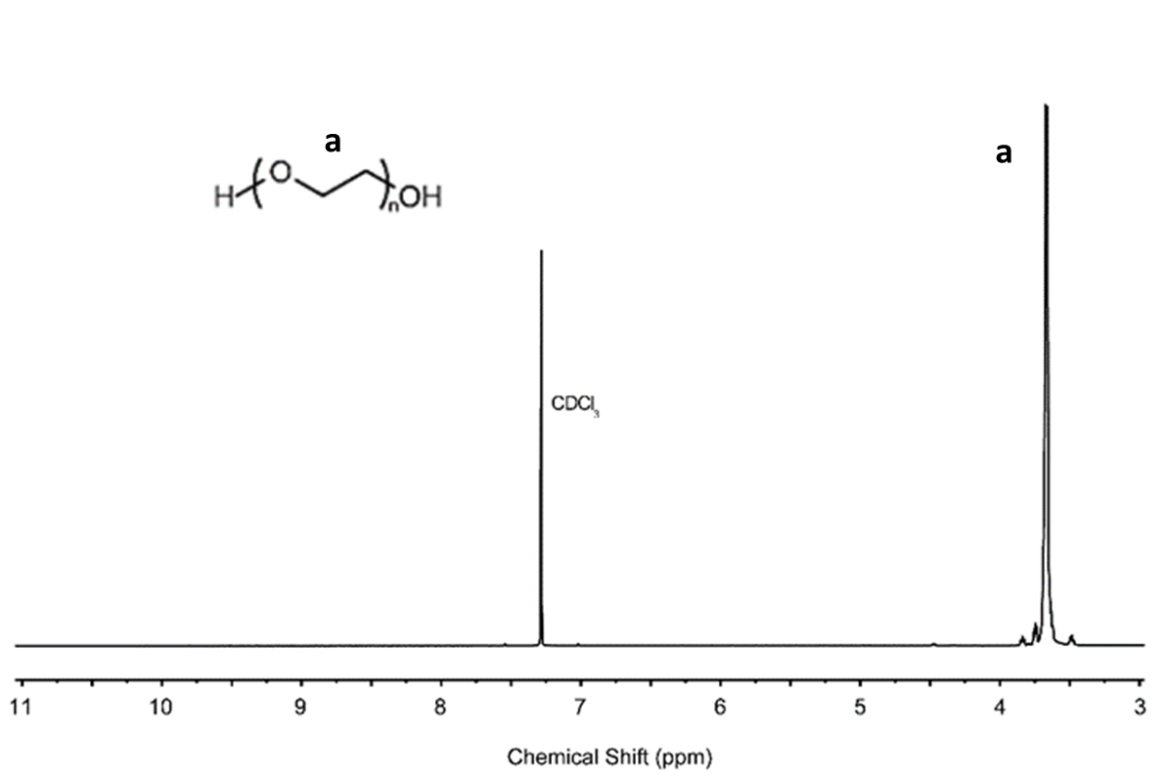


**Figure S1.** ^1^H NMR spectra of polyethylene glycol (PEG_4000_).


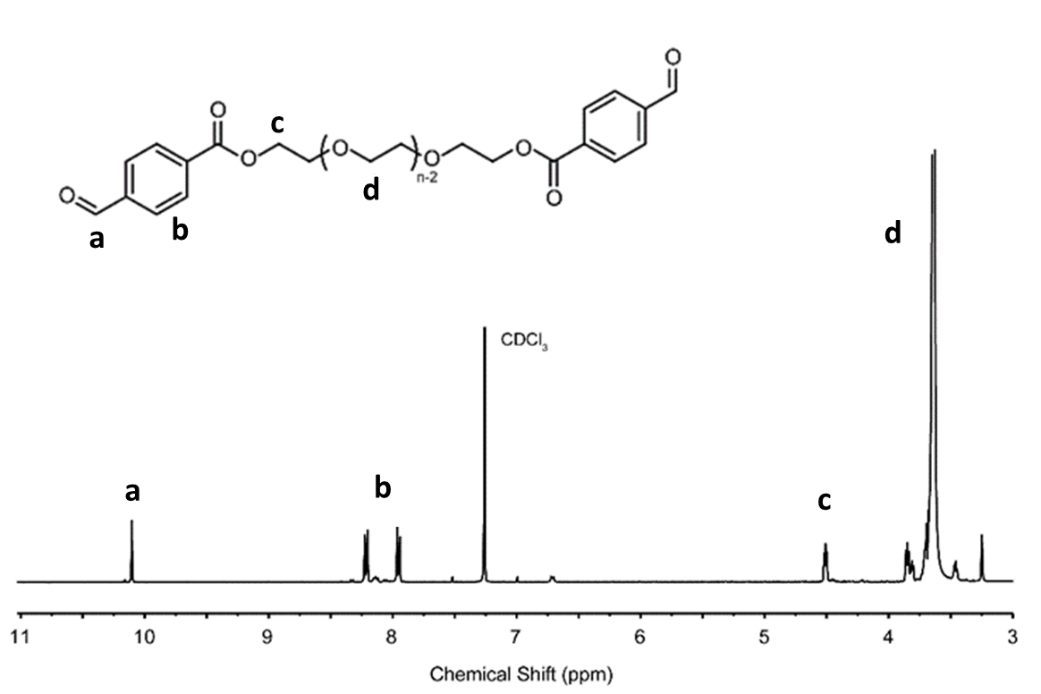


**Figure S2.** ^1^H NMR spectra of DF-PEG.


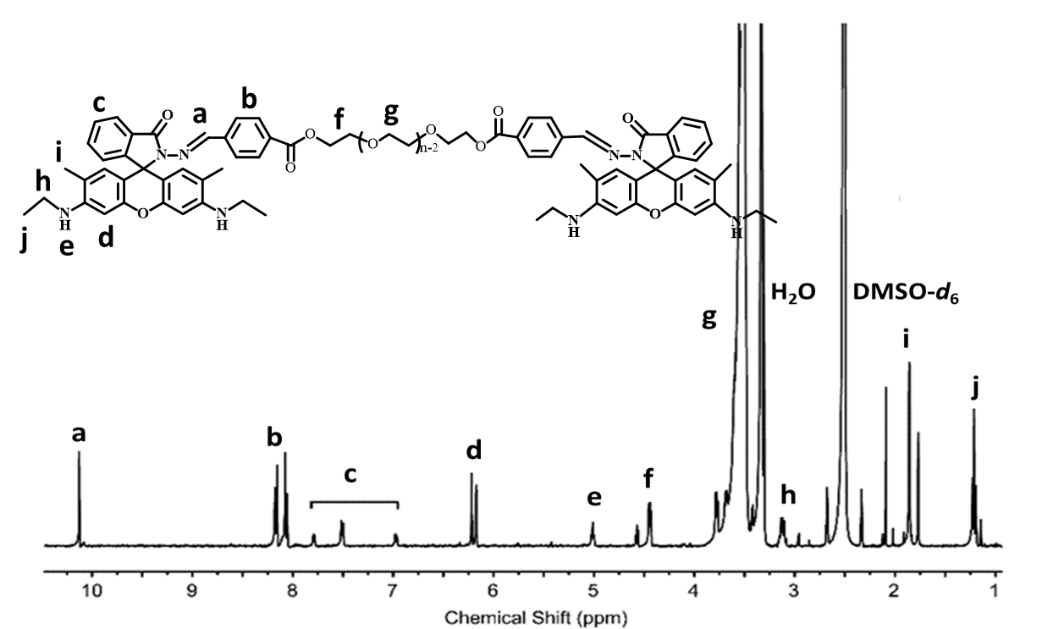


**Figure S3.** 1H NMR spectra of DRF-PEG.

**4. Images of DRF-PEG to Different Metal Ions**


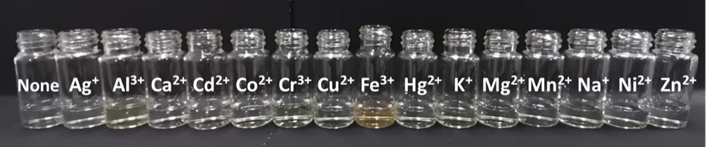


**Figure S4.** Images of DRF-PEG (0.1 mg/mL) in water in the presence of different metal ions (10^-4^ M). From left to right: None, Ag^+^, Al^3+^, Ca^2+^, Cd^2+^, Co^2+^, Cr^3+^, Cu^2+^, Fe^3+^, Hg^2+^, K^+^, Mg^2+^, Mn^2+^, Na^+^, Ni^2+^, and Zn^2+^.

**5. Enlarged CLSM Images of Hela Cells Treated with DRF-PEG**


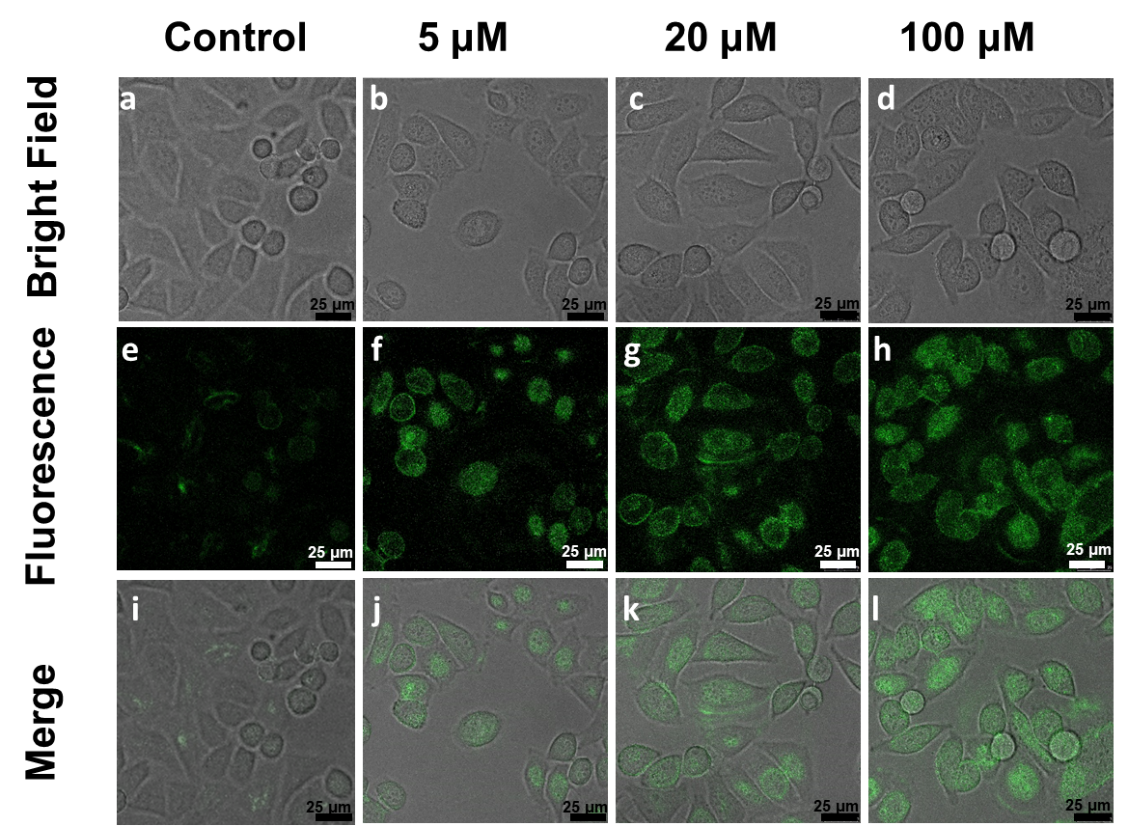


**Figure S5.** CLSM images of Hela cells treated with DRF-PEG (0.1 mg/mL) and different concentrations of Fe^3+^ (0, 5, 20, 100 μM).

**References**

Yang, X-F., Guo, X-Q., Zhao Y-B. (2002) Development of a novel rhodamine-type fluorescent probe to determine peroxynitrite. *Talanta* 57 (5): 883-90.

Park, TE., Lee, SH. (2020) A micellized fluorescence sensor based on amplified quenching for highly sensitive detection of non-transferrin-bound iron in serum. *Dalton Trans.* 49 (15): 46604.
